# Supplementary material for: Elementary integrate-and-fire process underlies pulse amplitudes in Electrodermal activity
Source: PLoS Comput Biol. 2021 Jul 7;17(7):e1009099. doi: 10.1371/journal.pcbi.1009099 (PMC8289084; doi:10.1371/journal.pcbi.1009099)
Supplement: S1 Appendix — Table A in S1 Appendix. Model fit results for awake and at rest cohort for Models 5–8. Bold and yellow background indicates best model according to AIC, bold and orange background indicates best model according to mean distance, bold and green background indicates best model according to maximum distance. Table B in S1 Appendix. Model fit results propofol sedation cohort for Models 5–8. Bold and yellow background indicates best model according to AIC, bold and orange background indicates best model according to mean distance, bold and green background indicates best model according to maximum distance. Fig A in S1 Appendix. QQ plots for Subject S1 from the awake and at rest cohort. Fig B in S1 Appendix. Rescaled QQ plots for Subject S1 from the awake and at rest cohort. Fig C in S1 Appendix. QQ plots for Subject S2 from the awake and at rest cohort. Fig D in S1 Appendix. Rescaled QQ plots for Subject S2 from the awake and at rest cohort. Fig E in S1 Appendix. QQ plots for Subject S3 from the awake and at rest cohort. Fig F in S1 Appendix. Rescaled QQ plots for Subject S3 from the awake and at rest cohort. Fig G in S1 Appendix. QQ plots for Subject S4 from the awake and at rest cohort. Fig H in S1 Appendix. Rescaled QQ plots for Subject S4 from the awake and at rest cohort. Fig I in S1 Appendix. QQ plots for Subject S5 from the awake and at rest cohort. Fig J in S1 Appendix. Rescaled QQ plots for Subject S5 from the awake and at rest cohort. Fig K in S1 Appendix. QQ plots for Subject S6 from the awake and at rest cohort. Fig L in S1 Appendix. Rescaled QQ plots for Subject S6 from the awake and at rest cohort. Fig M in S1 Appendix. QQ plots for Subject S7 from the awake and at rest cohort. Fig N in S1 Appendix. Rescaled QQ plots for Subject S7 from the awake and at rest cohort. Fig O in S1 Appendix. QQ plots for Subject S9 from the awake and at rest cohort. Fig P in S1 Appendix. Rescaled QQ plots for Subject S9 from the awake and at rest cohort. Fig Q in S1 Appendix. [file pcbi.1009099.s001.pdf]

S1 APPENDIX

Title: Elementary Integrate-and-Fire Process Underlies Pulse Amplitudes in Electrodermal Activity

Authors: Sandya Subramanian*, Patrick L. Purdon, Riccardo Barbieri, Emery N. Brown

*Corresponding Author

Corresponding Author E-mail: sandya@mit.edu

DETAILED RESULTS FOR MODELS 5-8

Table A. Model fit results for awake and at rest cohort for Models 5-8.

	3IG with known shift (Model 8)			Exponential (Model 7)			Gamma (Model 6)			Lognormal (Model 5)		
	AIC	Mean	Max	AIC	Mean	Max	AIC	Mean	Max	AIC	Mean	Max
S1	**-1098**	0.0024	0.0780	-1001	0.00160	0.0099	-1029	0.00204	0.0101	-1066	0.00162	0.0099
S2	-1073	0.0094	0.7340	-1729	0.00362	0.1721	-1747	0.00369	0.1864	**-1862**	**0.00239**	0.1723
S3	-604	0.0081	0.2520	-584	0.00425	0.0421	-584	0.00474	0.0504	-618	0.00307	0.0322
S4	-722	0.0135	0.4602	-707	0.00710	0.0641	-708	0.00830	0.0777	-757	0.00595	0.0449
S5	-714	0.0293	0.8779	-711	0.01419	0.1635	-797	0.00644	0.0661	-824	0.00439	0.1516
S6	-433	0.0137	0.3883	-529.4	0.00744	0.0487	-529.0	0.00691	0.0434	-573	0.00488	0.0470
S7	-388	0.0308	0.9522	-384	0.01320	0.1152	-382	0.01233	0.1053	-415	0.00907	0.0799
S8	-1073	0.0189	1.1360	-1012.8	0.01439	0.2308	-1013.3	0.01341	0.2128	-1127	0.00940	0.1605
S9	-1324	0.0154	0.7718	-1325	0.00540	0.1197	-1340	0.00662	0.1504	-1433	0.00499	0.0910
S10	-961	0.0019	0.0713	-932	0.00096	**0.0027**	-963	0.00109	0.0077	-1000	0.00089	0.0076
S11	-424	0.0267	0.9003	-445	0.00696	0.0563	-444	0.00596	0.0511	-467	0.00624	0.1423

Bold and yellow background indicates best model according to AIC, bold and orange background indicates best model according to mean distance, bold and green background indicates best model according to maximum distance.

Table B. Model fit results propofol sedation cohort for Models 5-8.

	3IG with known shift (Model 8)			Exponential (Model 7)			Gamma (Model 6)			Lognormal (Model 5)		
	AIC	Mean	Max	AIC	Mean	Max	AIC	Mean	Max	AIC	Mean	Max
P1	1452	0.4444	65.0399	562	0.15458	1.5962	454	0.09996	0.9653	222	0.0946	6.6980
P2	-718	0.0471	3.6942	-917	0.02982	1.5101	-915	0.02969	1.5051	-1106	0.0185	1.3319
P3	970	0.3081	40.4074	881	0.26527	14.9232	678	0.20785	13.3930	282	0.1187	7.3166
P4	351	0.1384	29.9697	-1056	0.08458	6.6325	-1147	0.07442	6.3296	-1767	0.0527	5.4607
P5	815	0.3357	26.0754	1092	0.34848	18.0306	971	0.27206	16.1510	707	0.1471	8.1513
P6	-1165	0.1020	16.3274	-1353	0.06431	1.0177	-1443	0.05423	0.8276	-1923	0.0420	0.6758
P7	53	0.1860	29.9329	49	0.15824	13.9430	-166	0.13130	13.1840	-925	0.0875	10.7545
P8	-106	0.1273	12.3888	-89	0.14318	9.5849	-145	0.12754	9.1268	-491	0.0938	8.5924
P9	-904	0.0658	4.4293	-644	0.04718	9.2322	-660	0.04778	9.3685	**-990**	0.0406	9.2512
P10	-1820	0.0220	1.8786	-2288	0.01339	0.5838	-2398	0.01205	0.6731	**-2695**	0.0091	0.6482
P11	-636	0.0970	7.9576	-896	0.06640	6.0904	-916	0.06031	5.9448	-1335	0.0452	5.2760

Bold and yellow background indicates best model according to AIC, bold and orange background indicates best model according to mean distance, bold and green background indicates best model according to maximum distance.

AWAKE AND AT REST COHORT

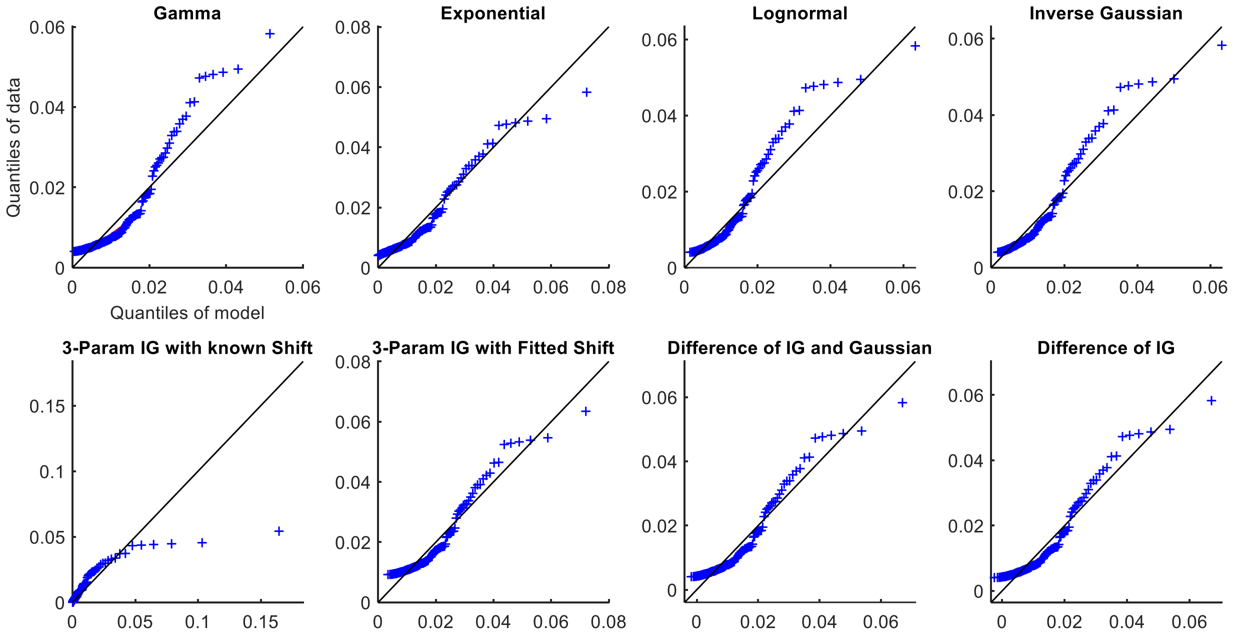

Fig A. QQ plots for Subject S1 from the awake and at rest cohort

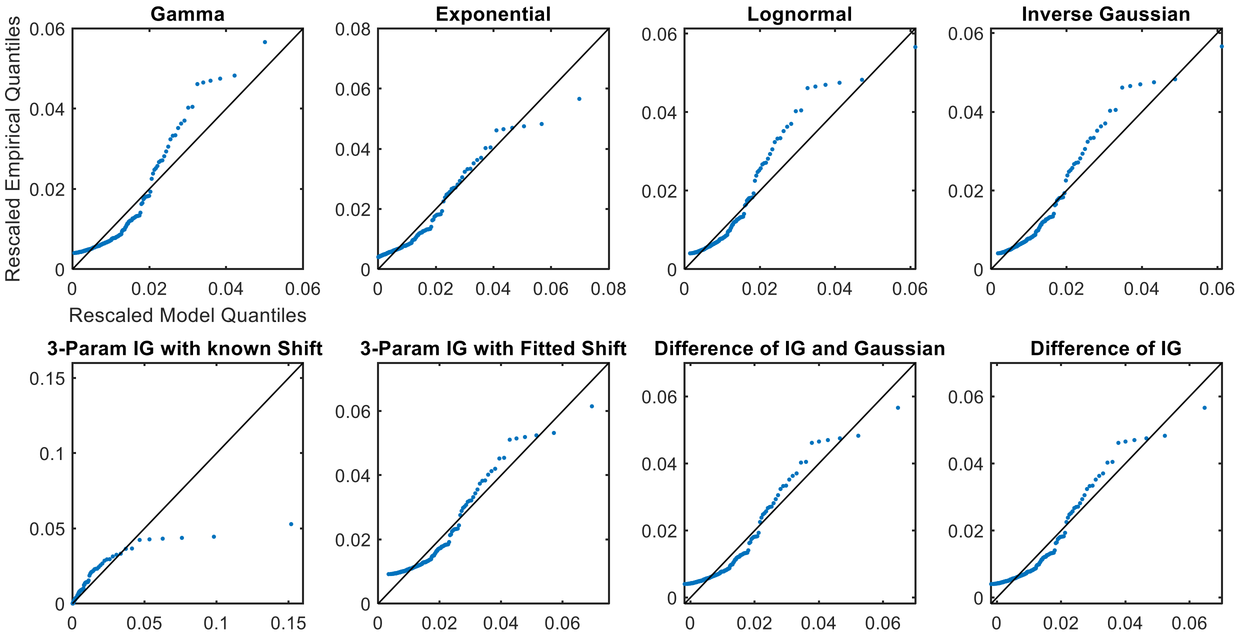

Fig B. Rescaled QQ plots for Subject S1 from the awake and at rest cohort

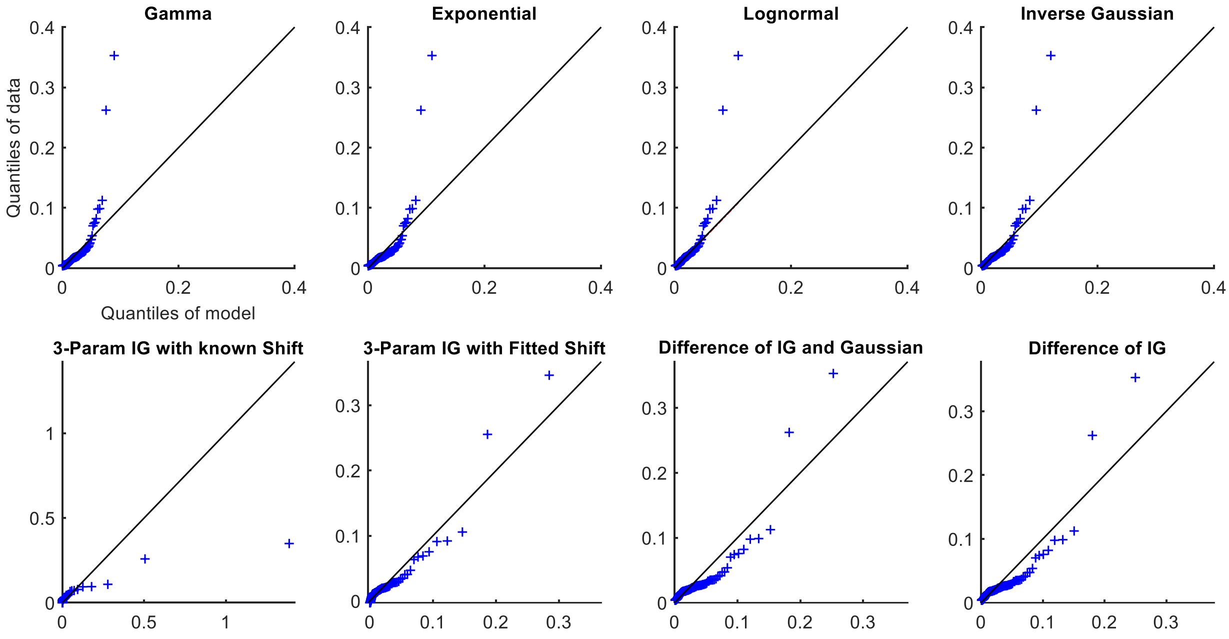

Fig C. QQ plots for Subject S2 from the awake and at rest cohort

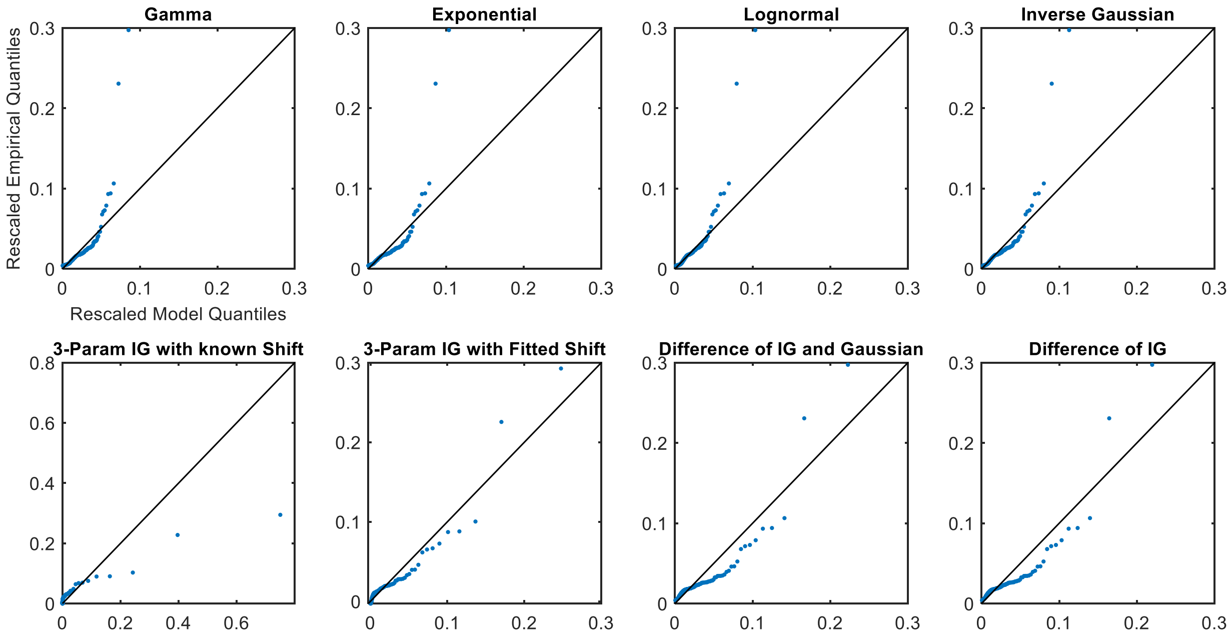

Fig D. Rescaled QQ plots for Subject S2 from the awake and at rest cohort

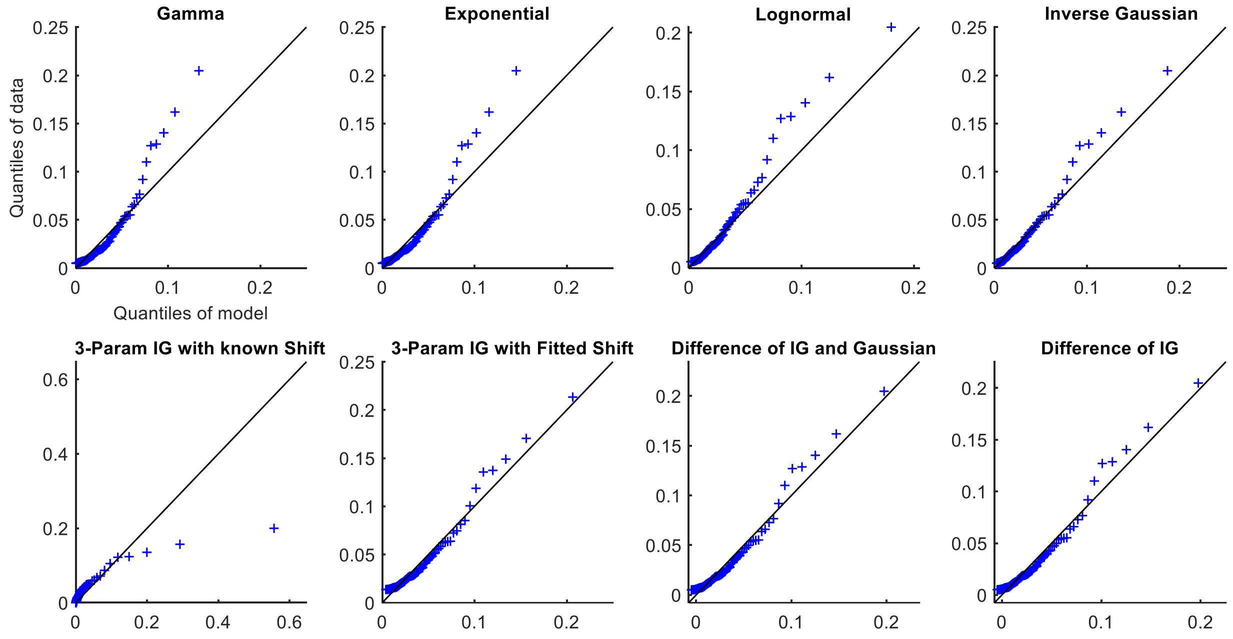

Fig E. QQ plots for Subject S3 from the awake and at rest cohort

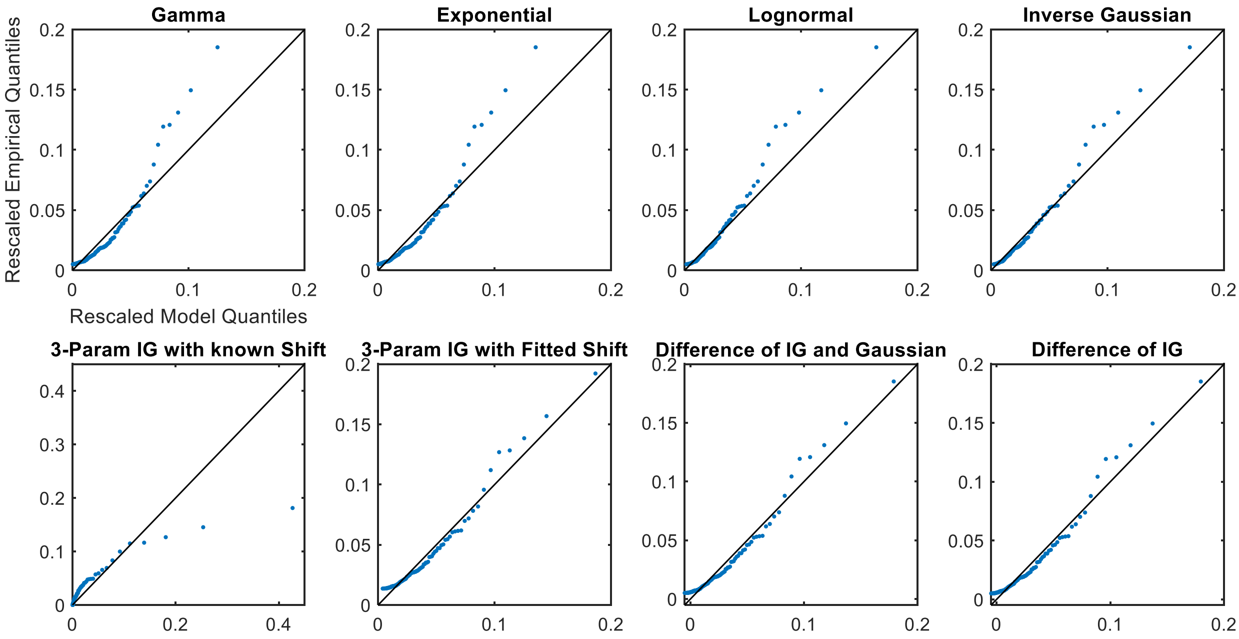

Fig F. Rescaled QQ plots for Subject S3 from the awake and at rest cohort

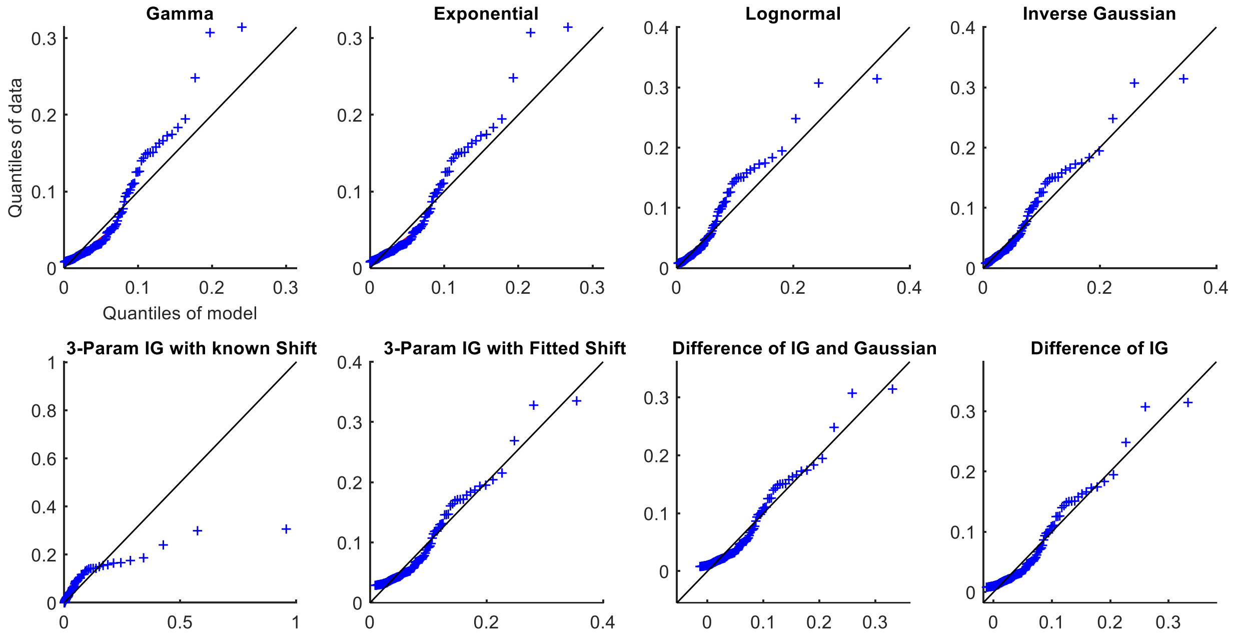

Fig G. QQ plots for Subject S4 from the awake and at rest cohort

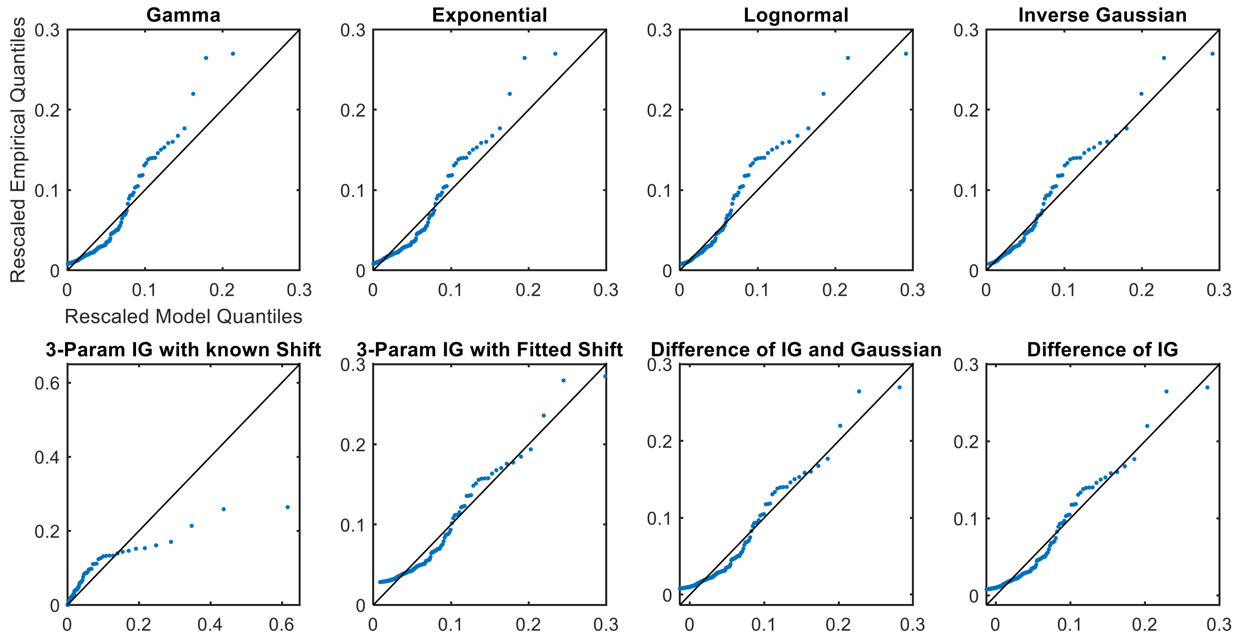

Fig H. Rescaled QQ plots for Subject S4 from the awake and at rest cohort

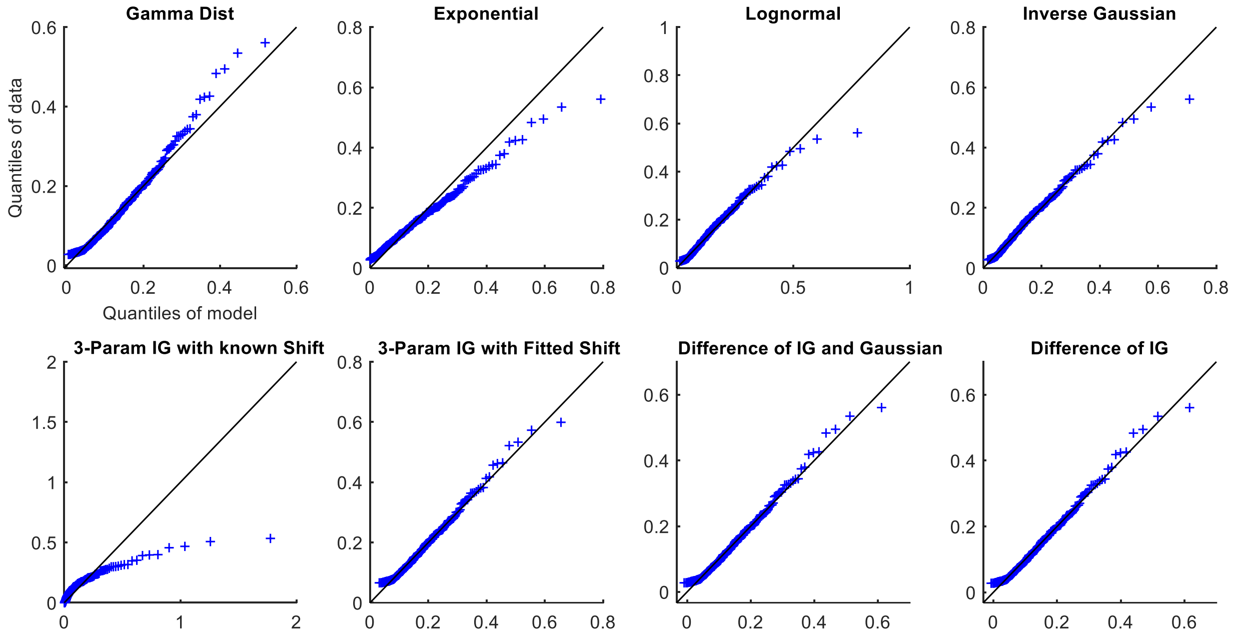

Fig I. QQ plots for Subject S5 from the awake and at rest cohort

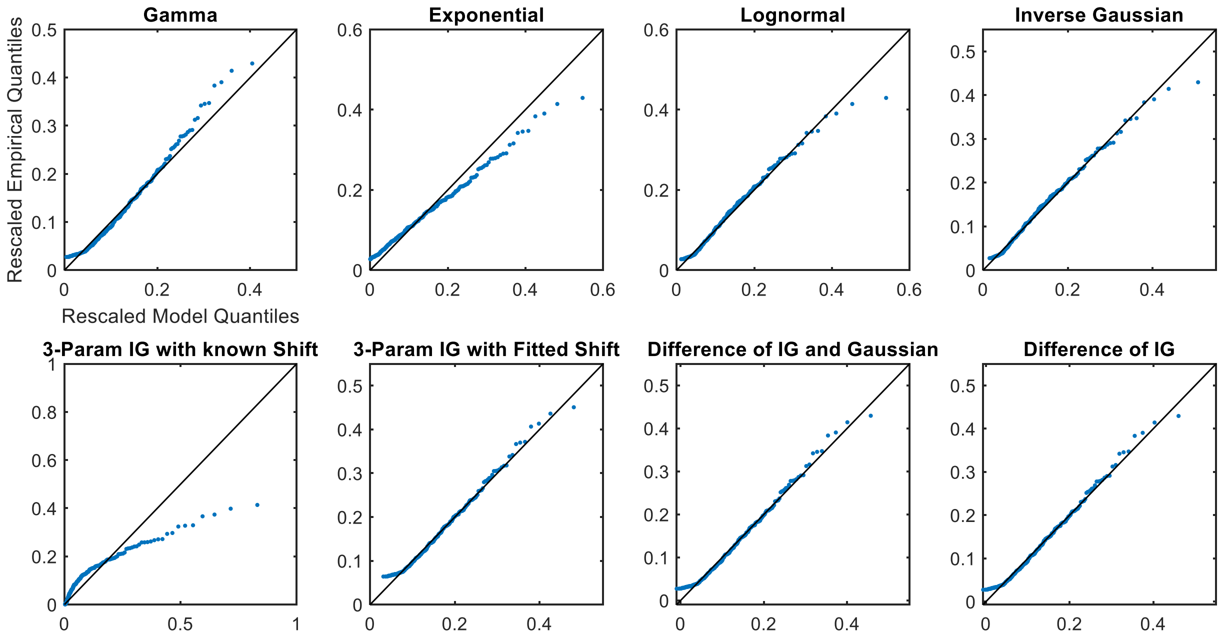

Fig J. Rescaled QQ plots for Subject S5 from the awake and at rest cohort

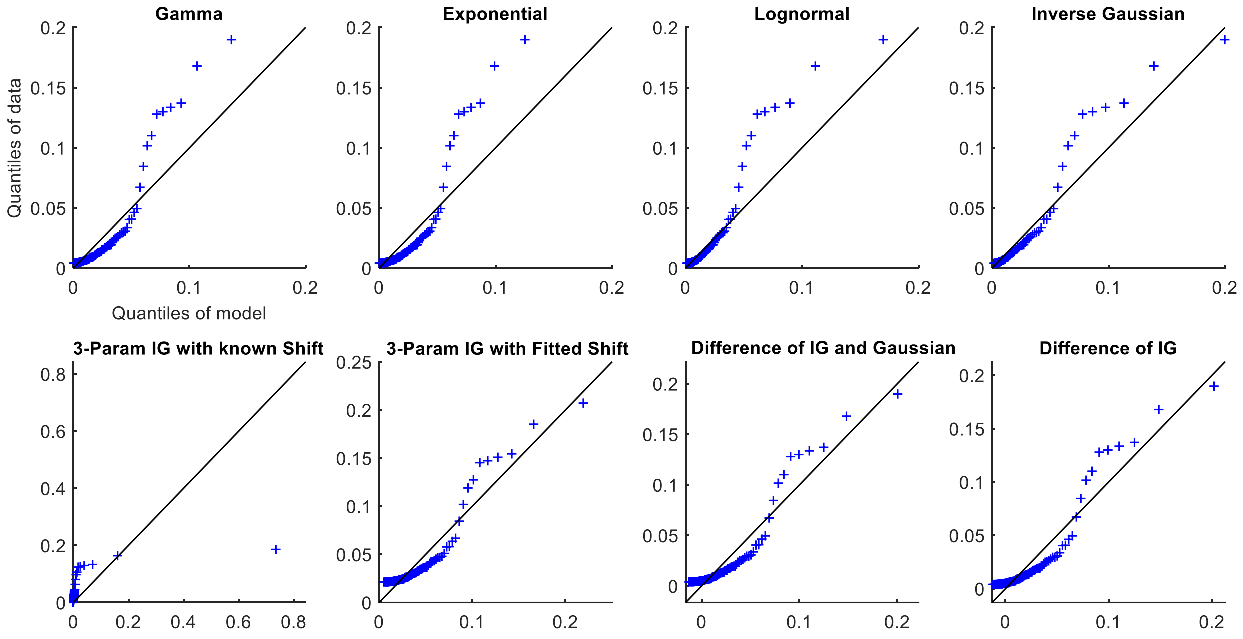

Fig K. QQ plots for Subject S6 from the awake and at rest cohort

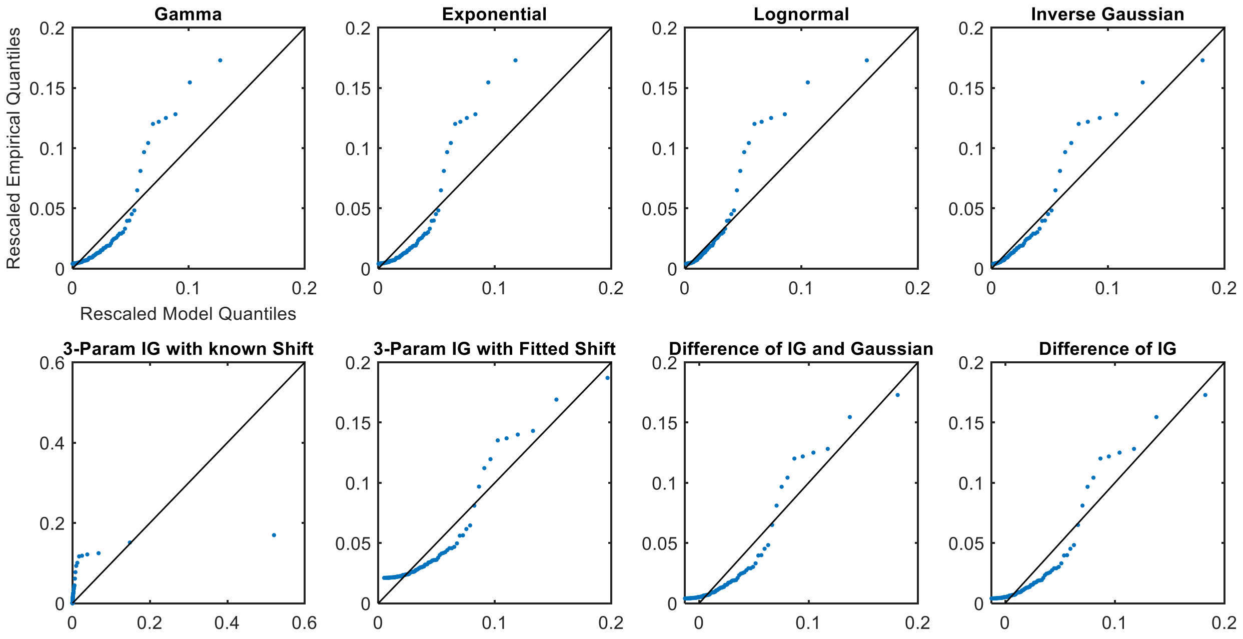

Fig L. Rescaled QQ plots for Subject S6 from the awake and at rest cohort

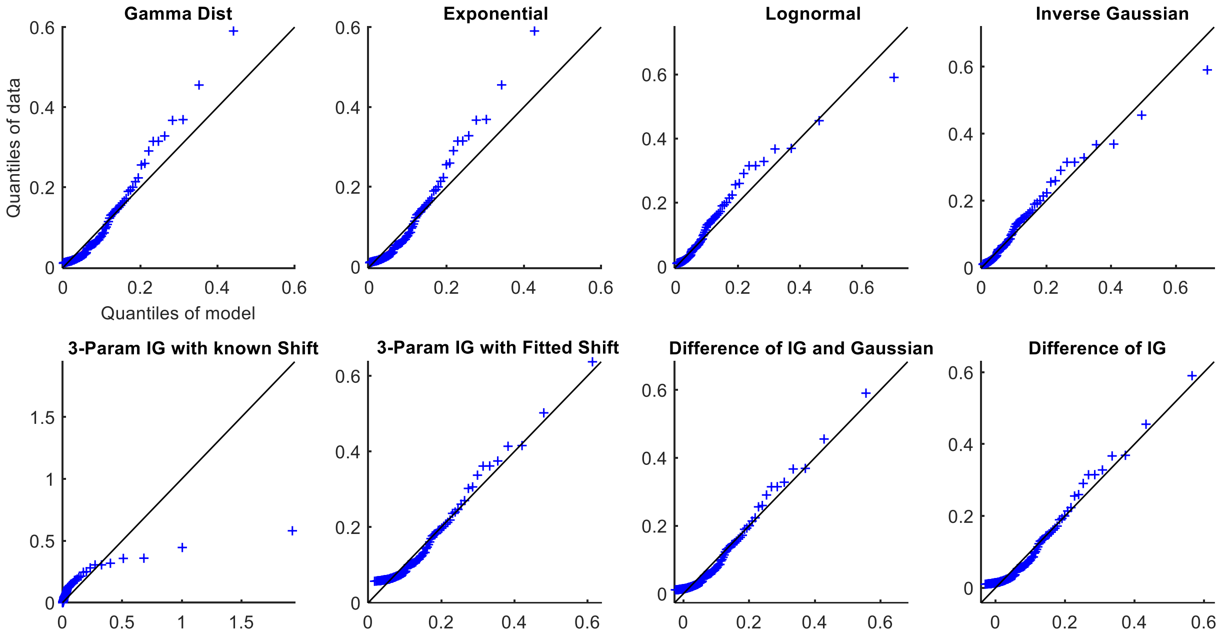

Fig M. QQ plots for Subject S7 from the awake and at rest cohort

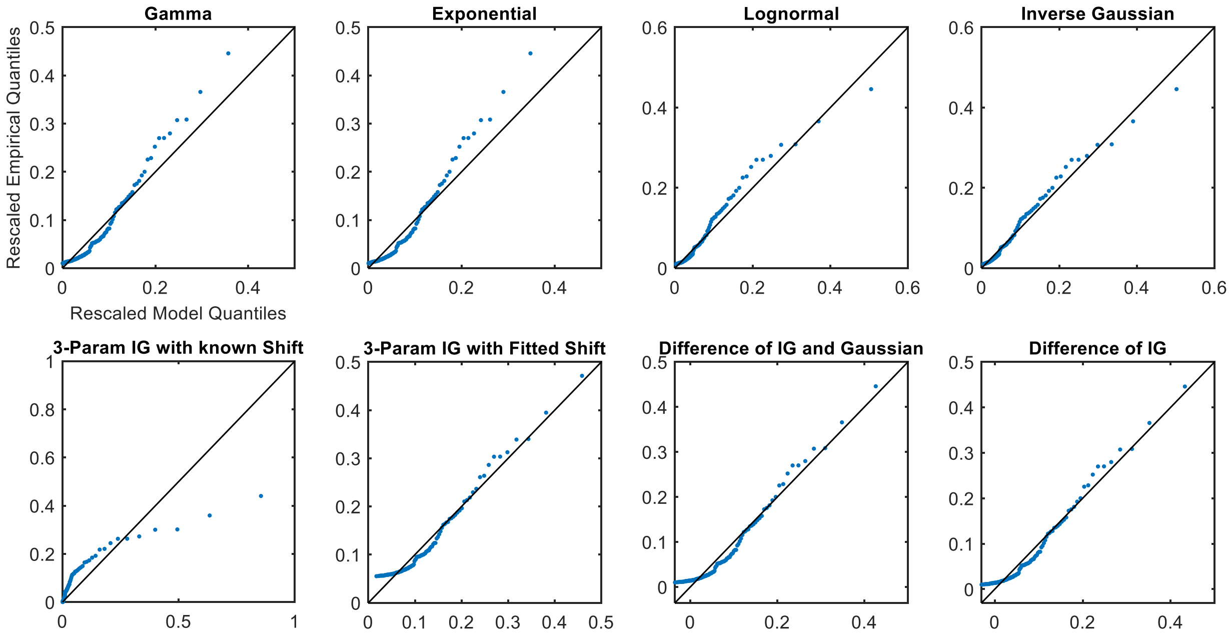

Fig N. Rescaled QQ plots for Subject S7 from the awake and at rest cohort

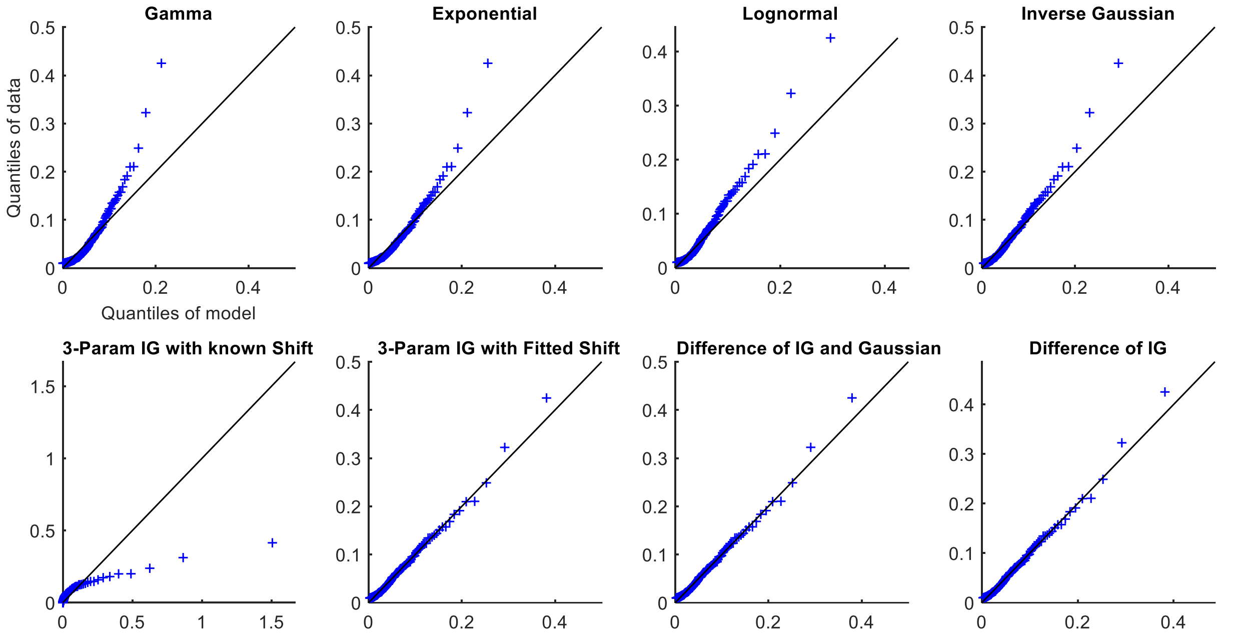

Fig O. QQ plots for Subject S9 from the awake and at rest cohort

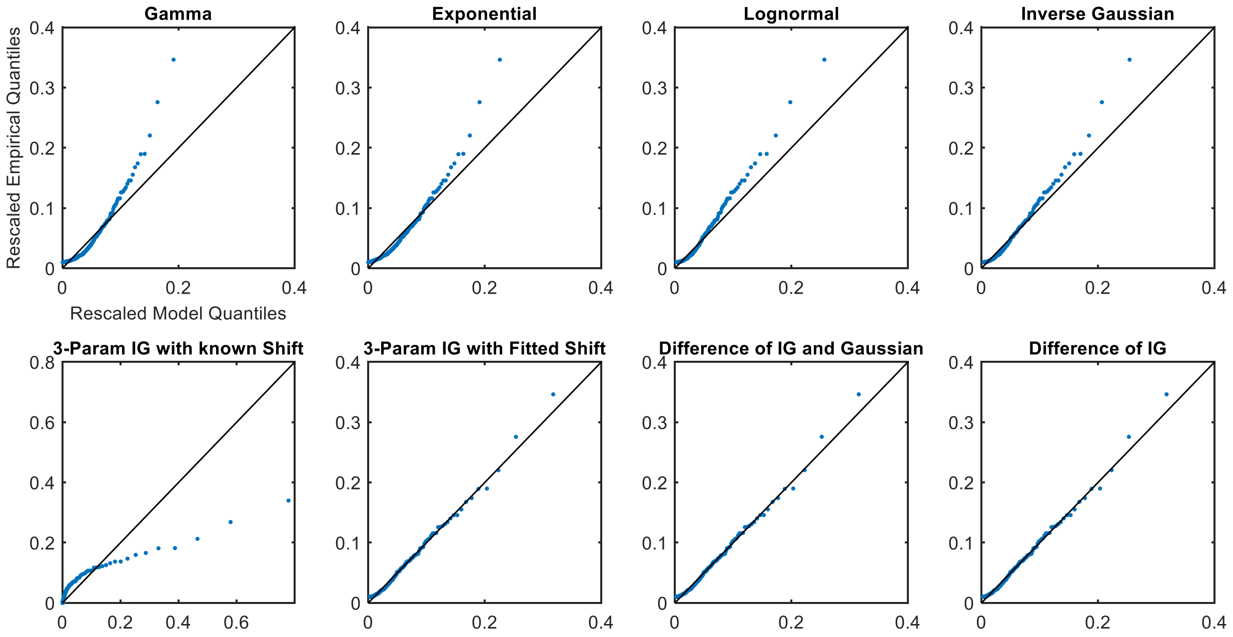

Fig P. Rescaled QQ plots for Subject S9 from the awake and at rest cohort

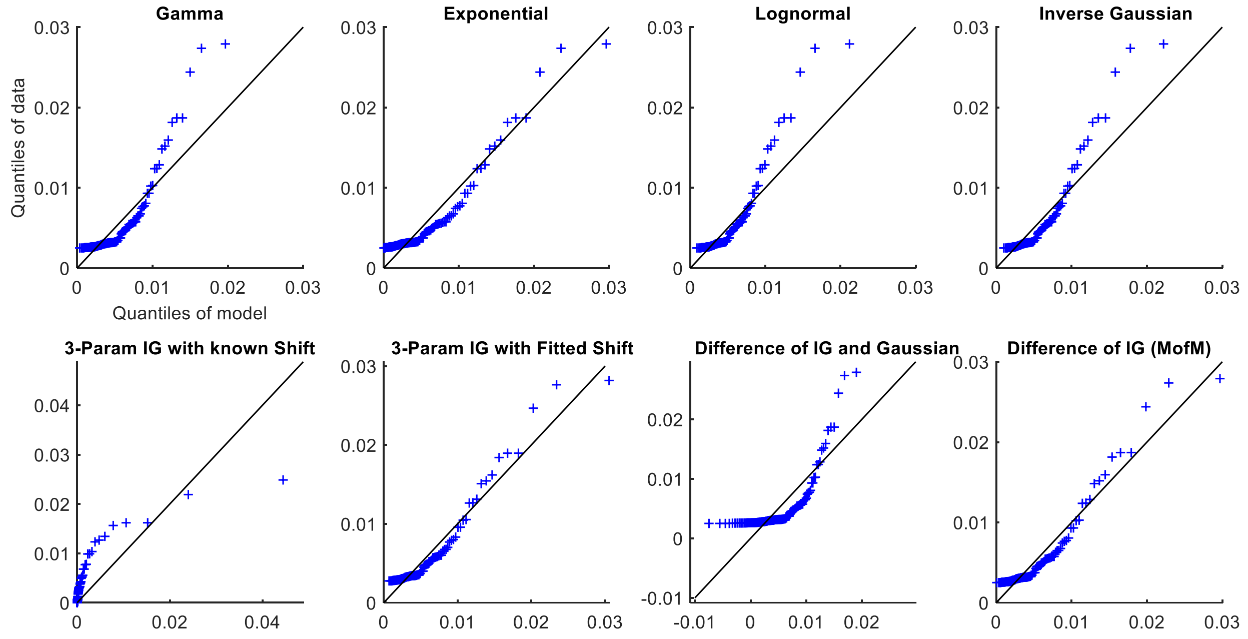

Fig Q. QQ plots for Subject S10 from the awake and at rest cohort

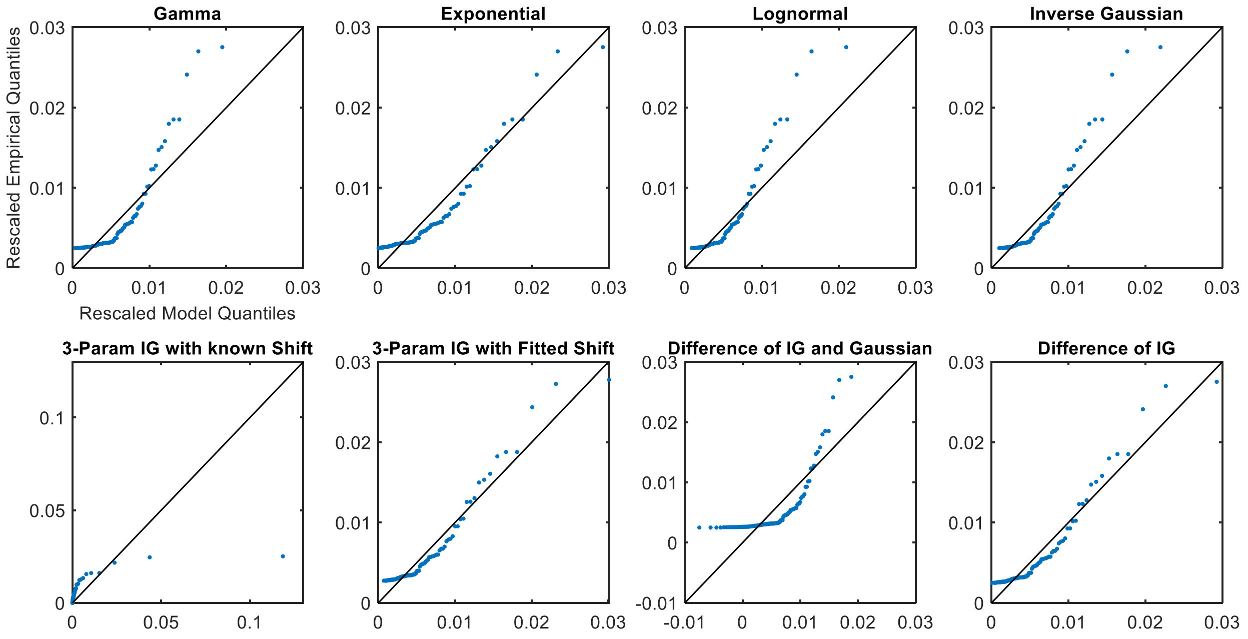

Fig R. Rescaled QQ plots for Subject S10 from the awake and at rest cohort

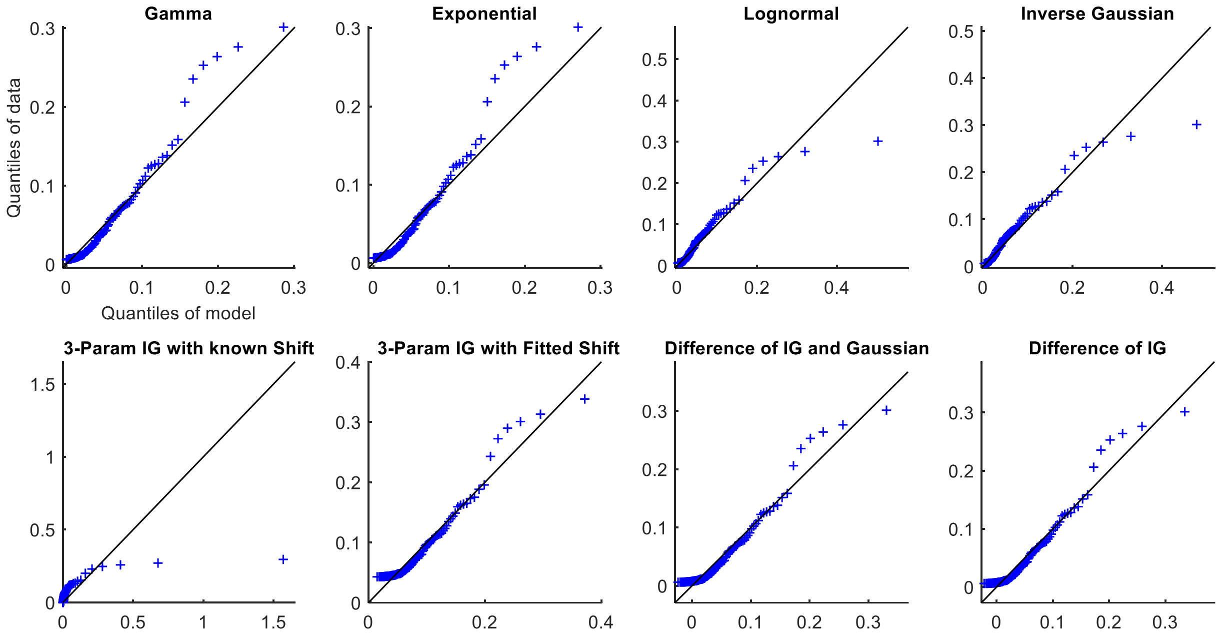

Fig S. QQ plots for Subject S11 from the awake and at rest cohort

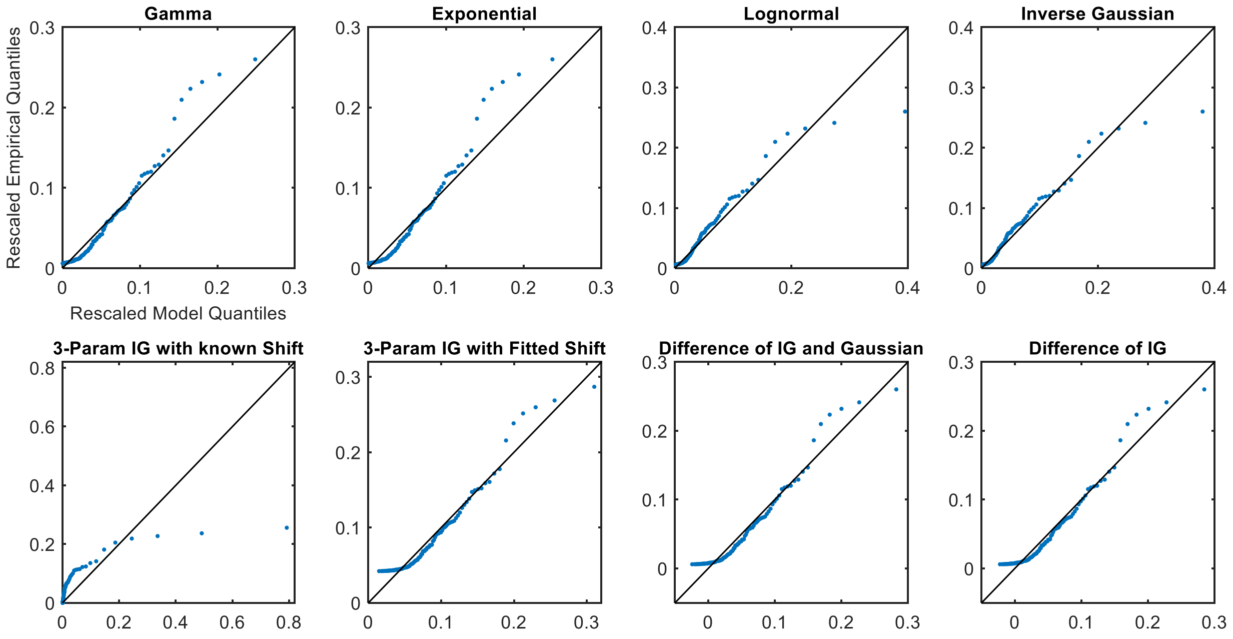

Fig T. Rescaled QQ plots for Subject S11 from the awake and at rest cohort

PROPOFOL SEDATION COHORT

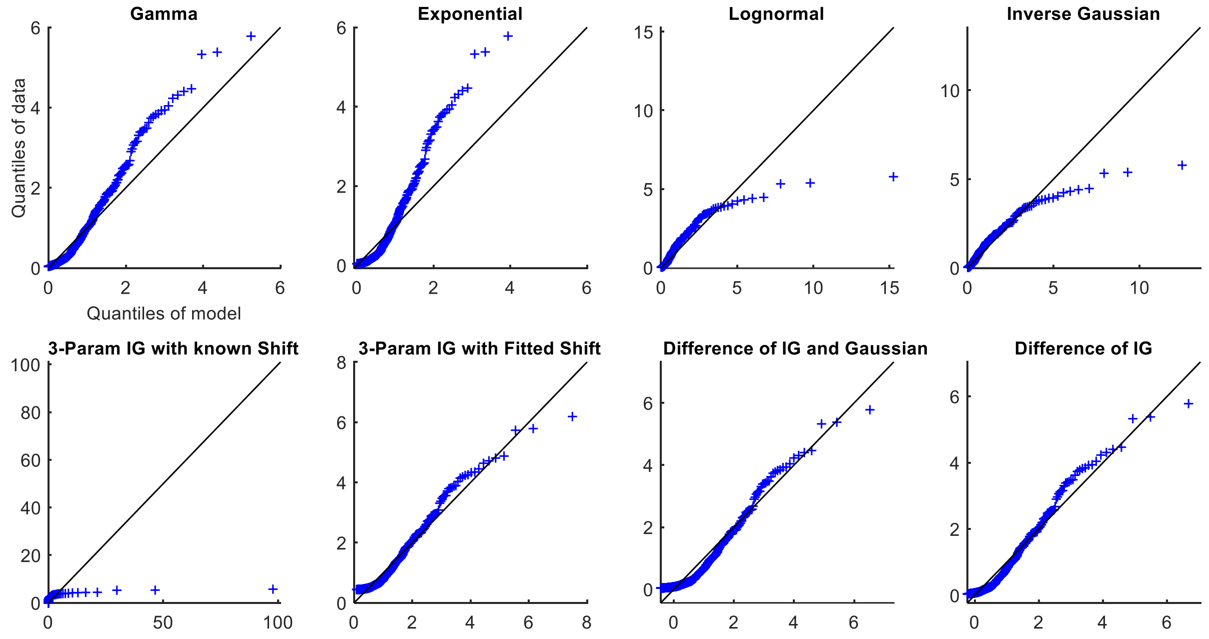

Fig U. QQ plots for Subject P1 from the propofol sedation cohort

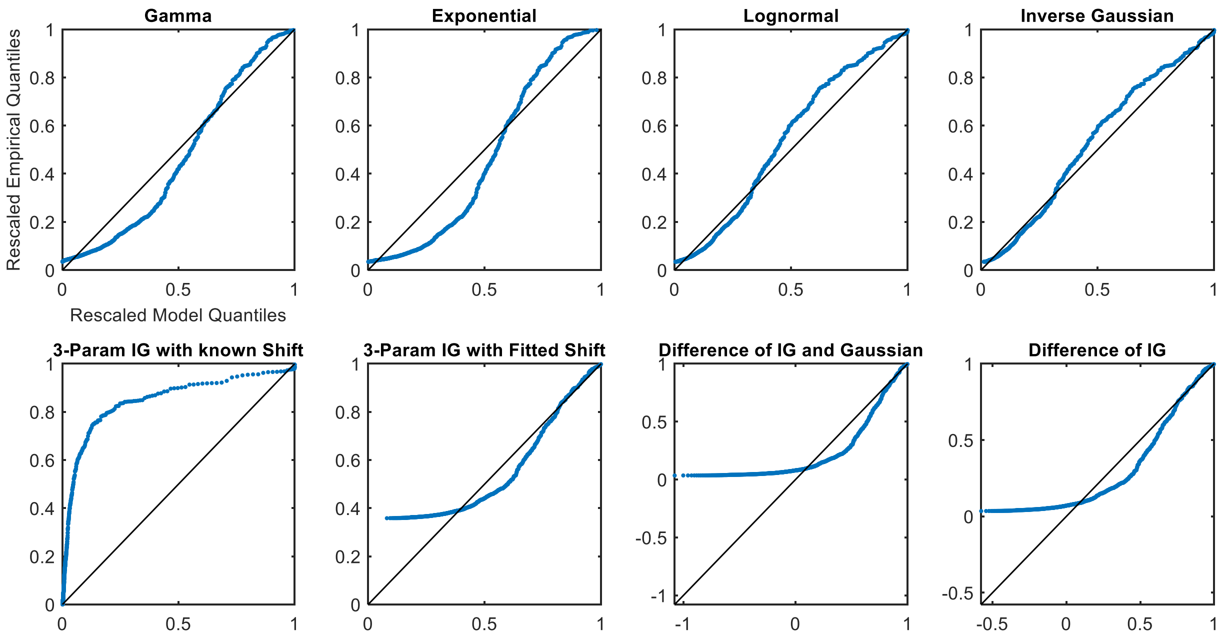

Fig V. Rescaled QQ plots for Subject P1 from the propofol sedation cohort

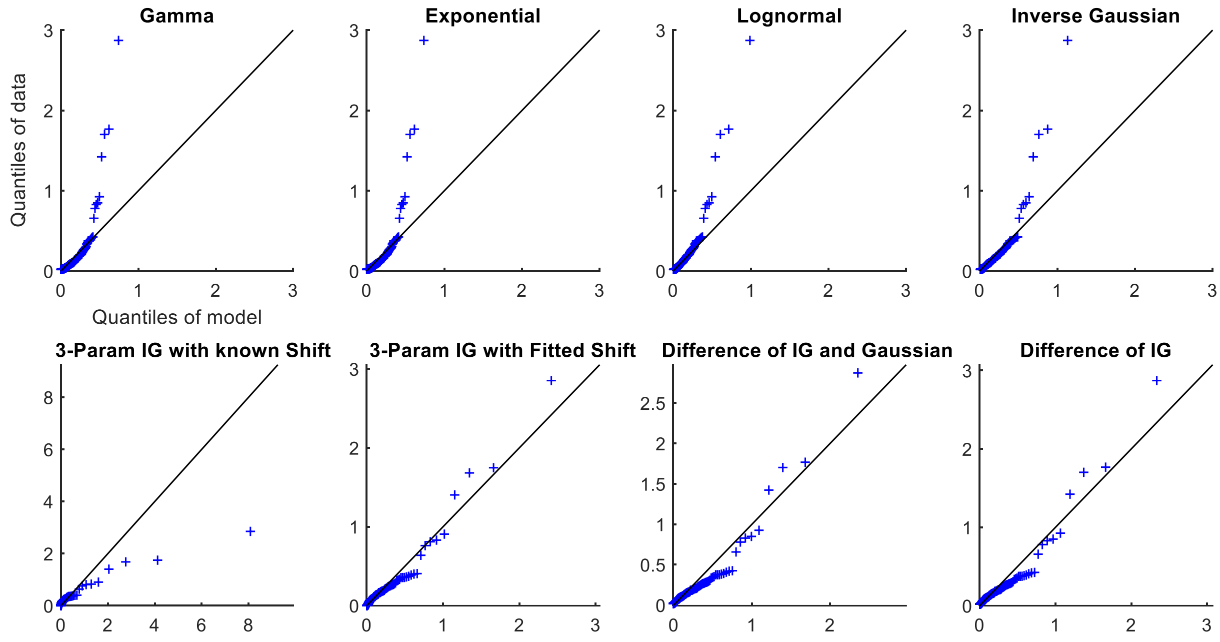

Fig W. QQ plots for Subject P2 from the propofol sedation cohort

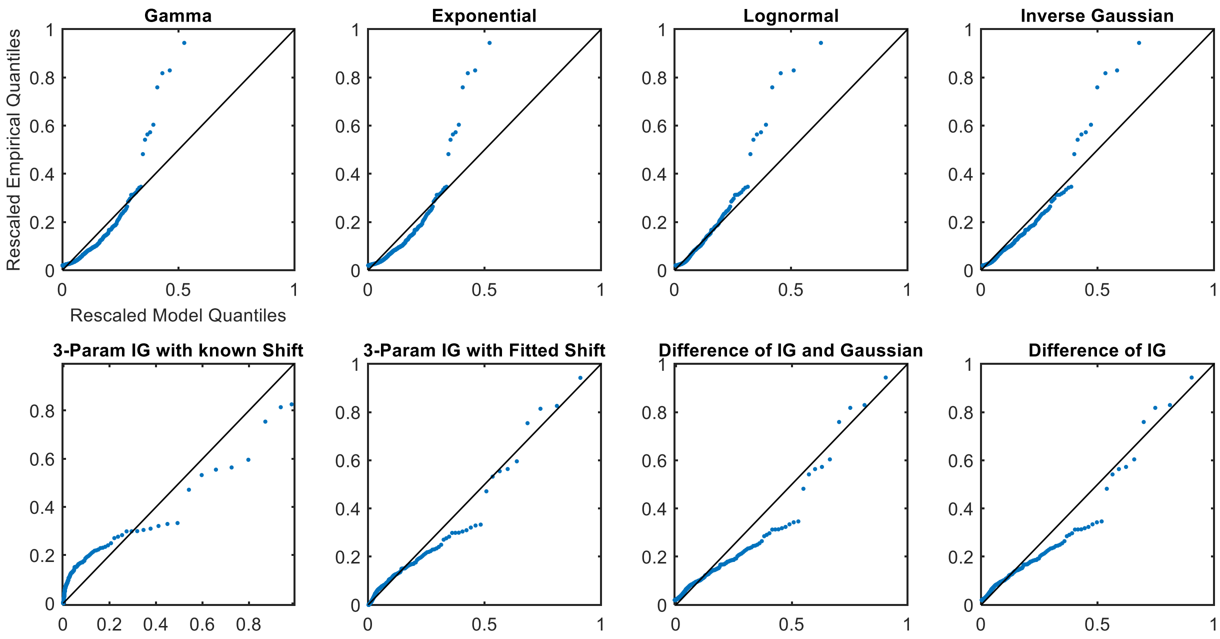

Fig X. Rescaled QQ plots for Subject P2 from the propofol sedation cohort

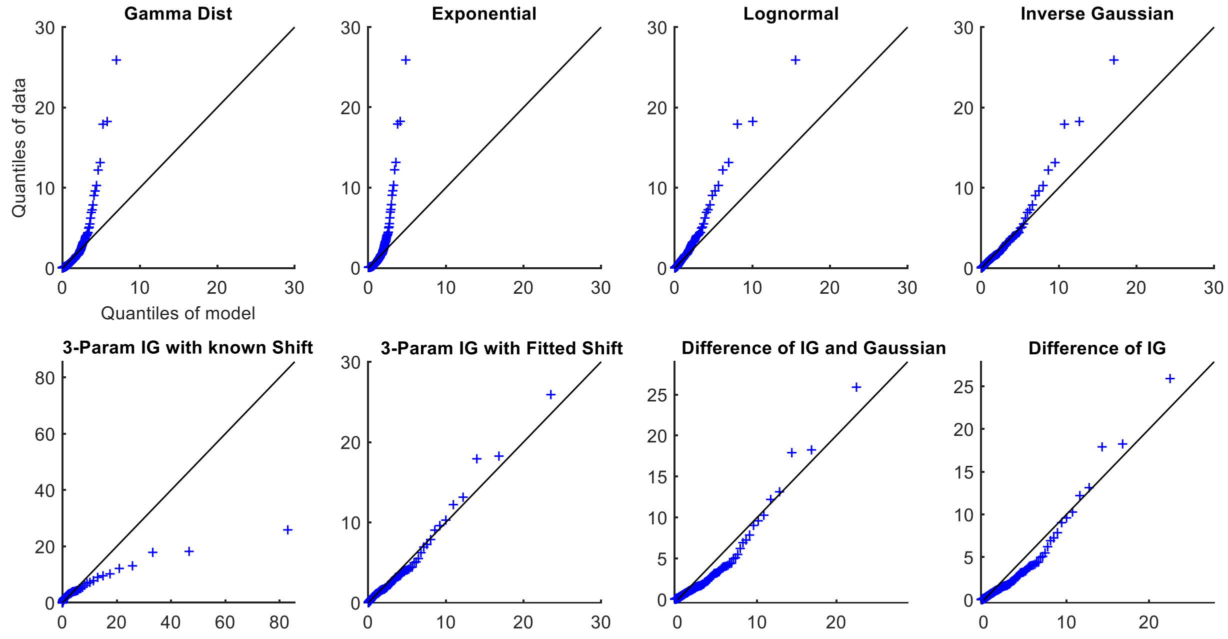

Fig Y. QQ plots for Subject P3 from the propofol sedation cohort

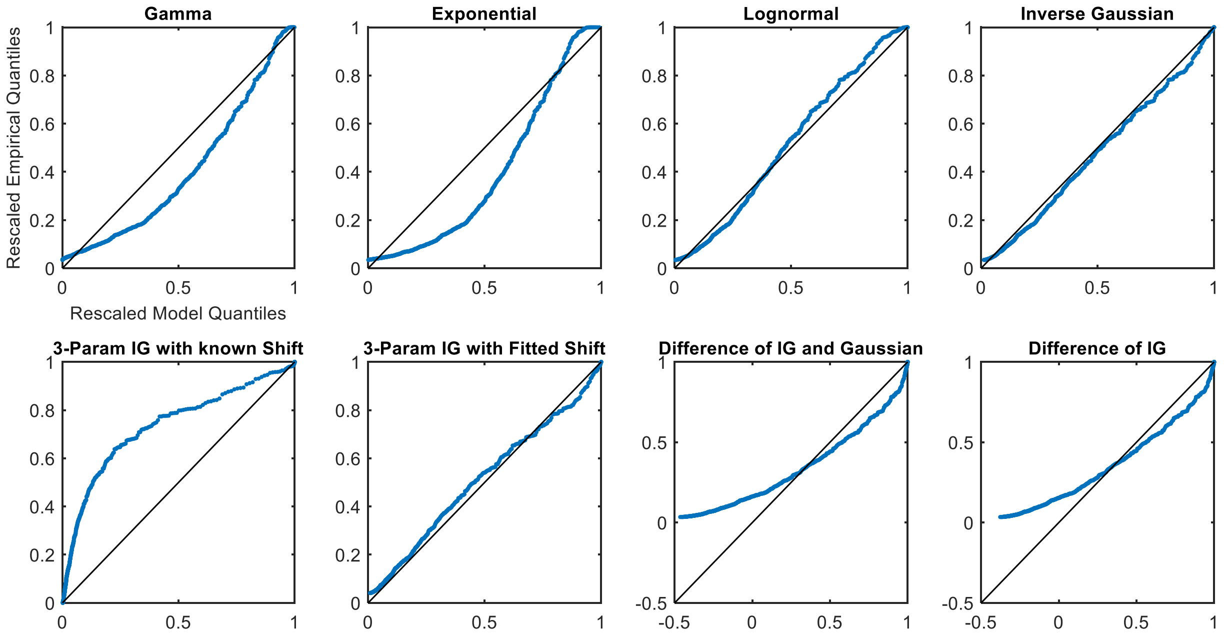

Fig Z. Rescaled QQ plots for Subject P3 from the propofol sedation cohort

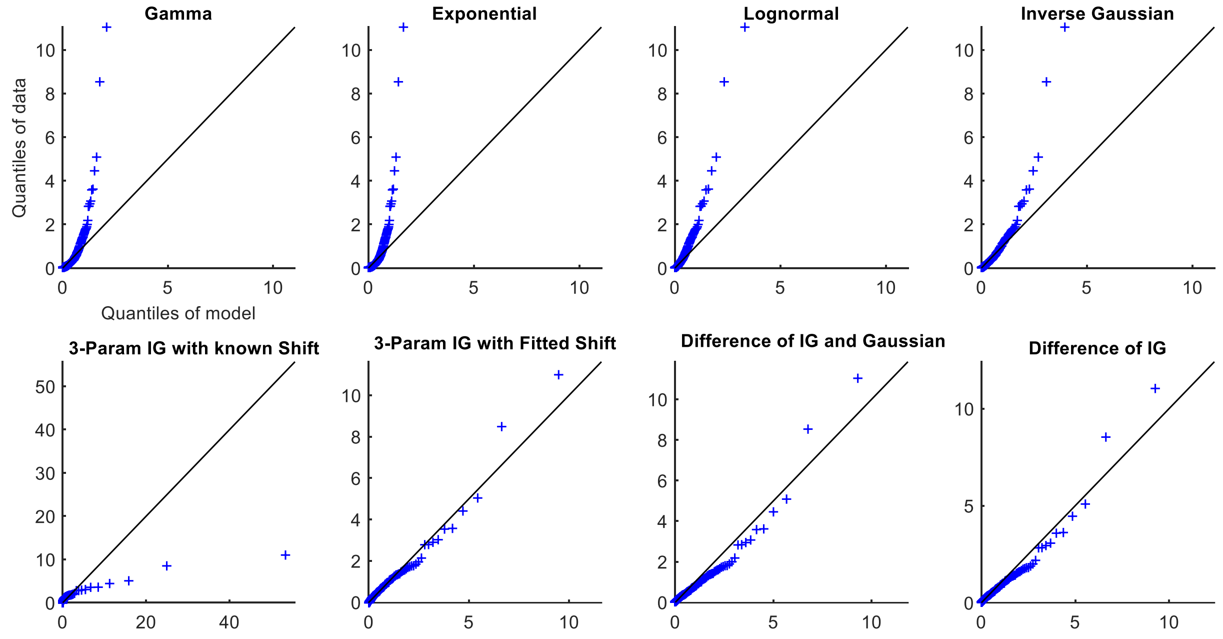

Fig AA. QQ plots for Subject P4 from the propofol sedation cohort

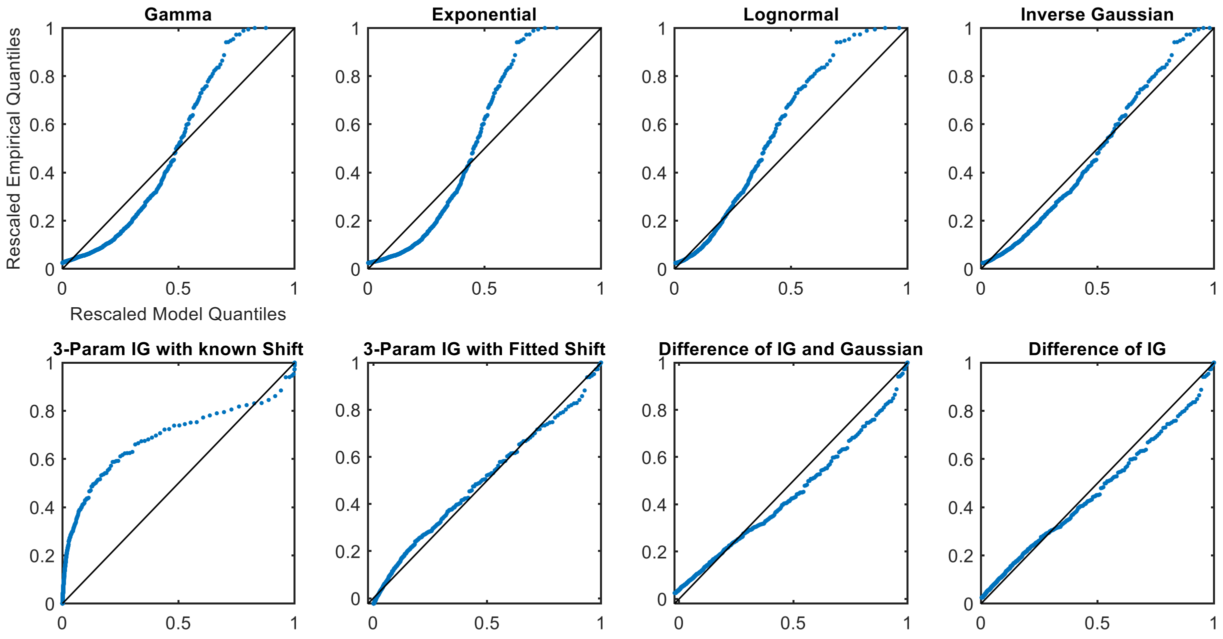

Fig AB. Rescaled QQ plots for Subject P4 from the propofol sedation cohort

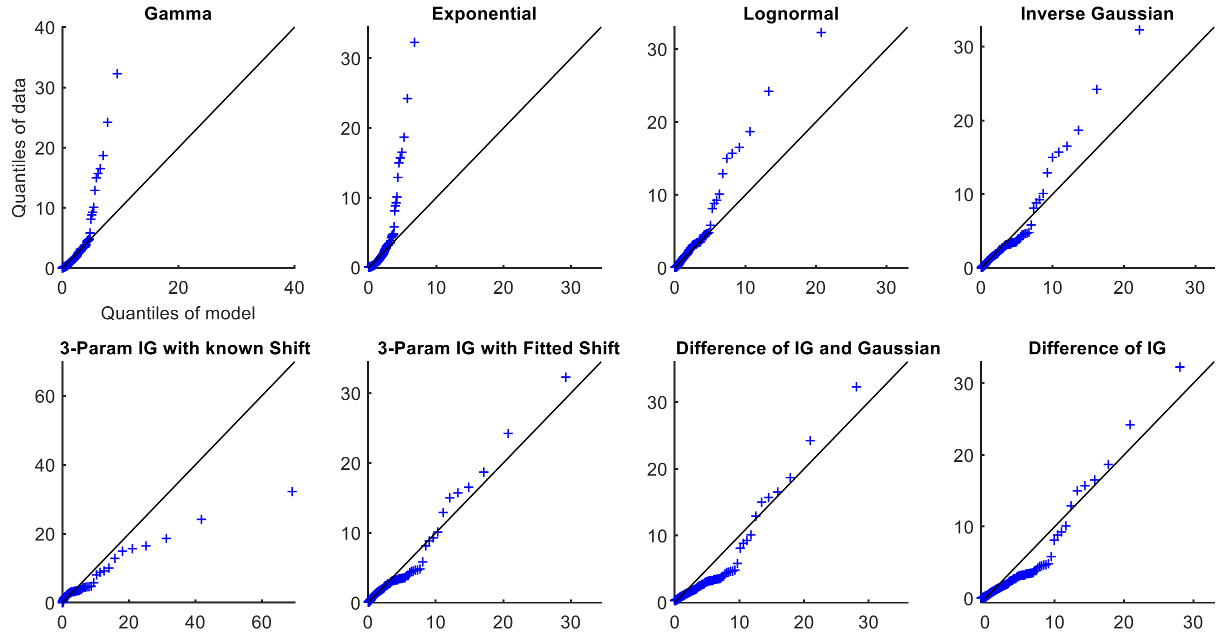

Fig AC. QQ plots for Subject P5 from the propofol sedation cohort

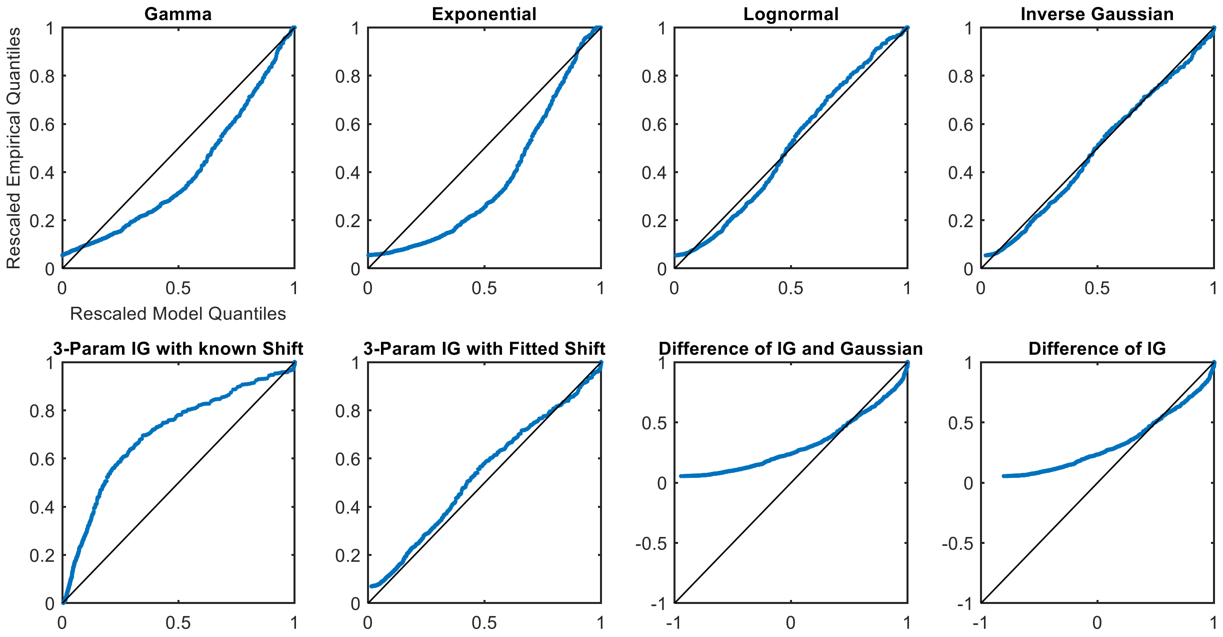

Fig AD. Rescaled QQ plots for Subject P5 from the propofol sedation cohort

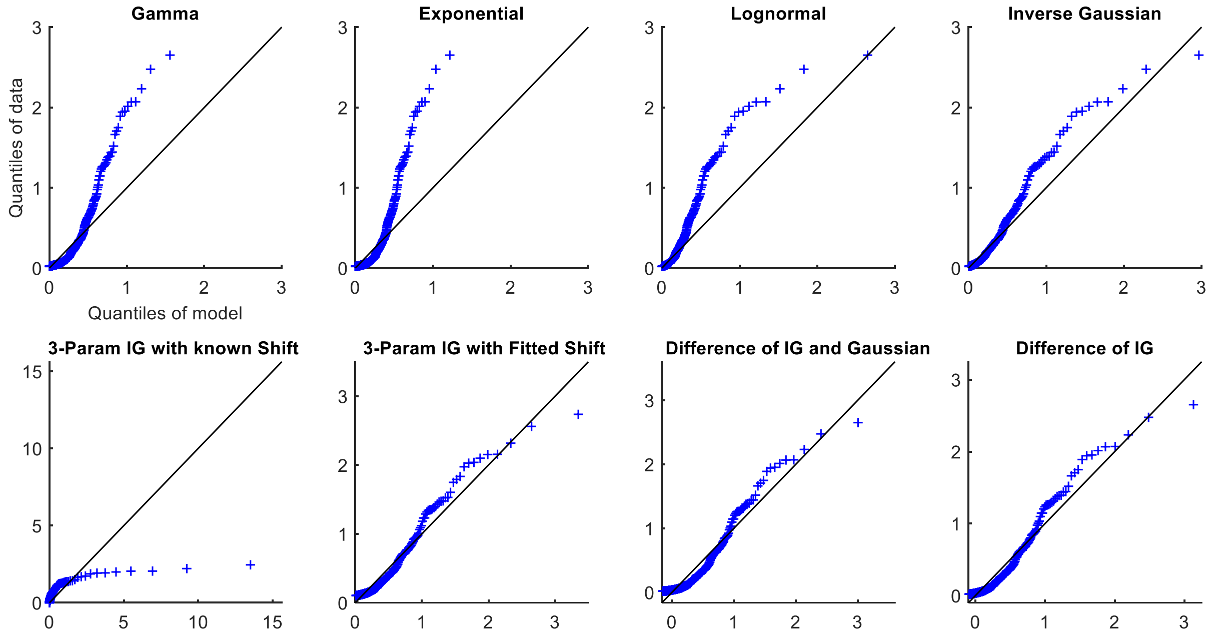

Fig AE. QQ plots for Subject P6 from the propofol sedation cohort

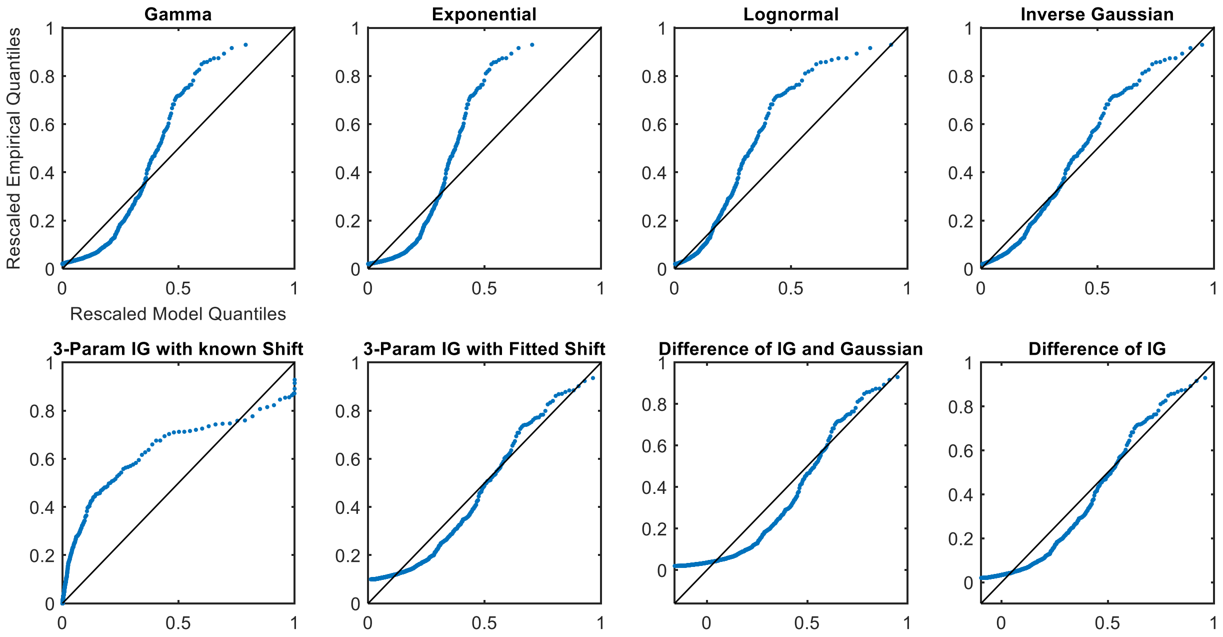

Fig AF. Rescaled QQ plots for Subject P6 from the propofol sedation cohort

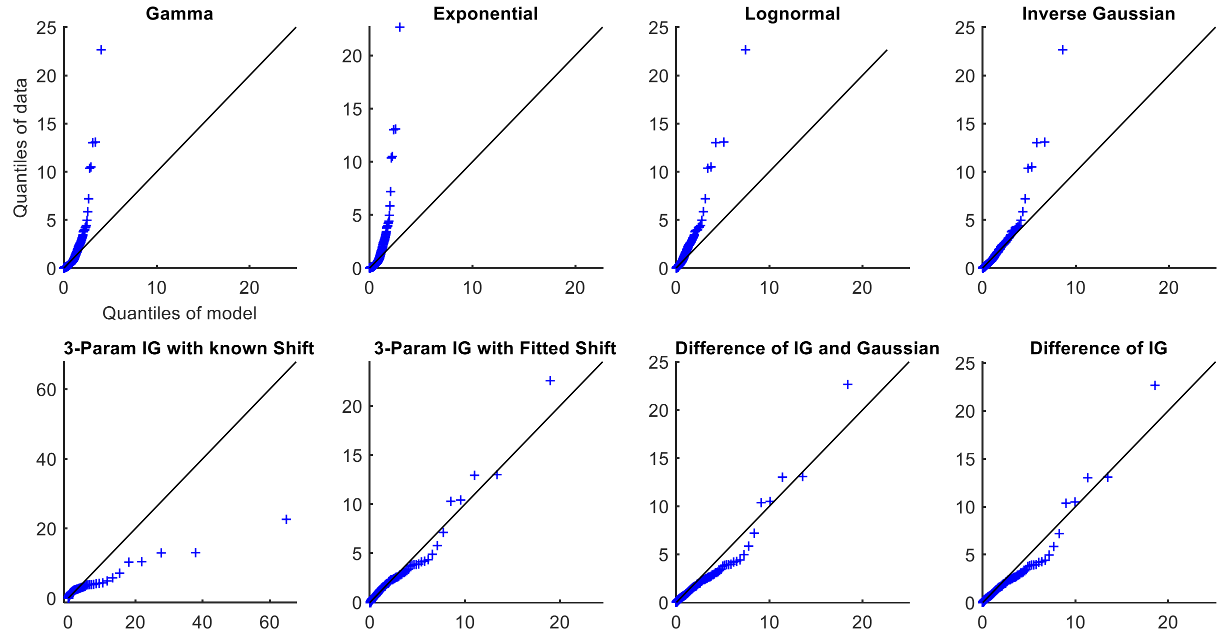

Fig AG. QQ plots for Subject P7 from the propofol sedation cohort

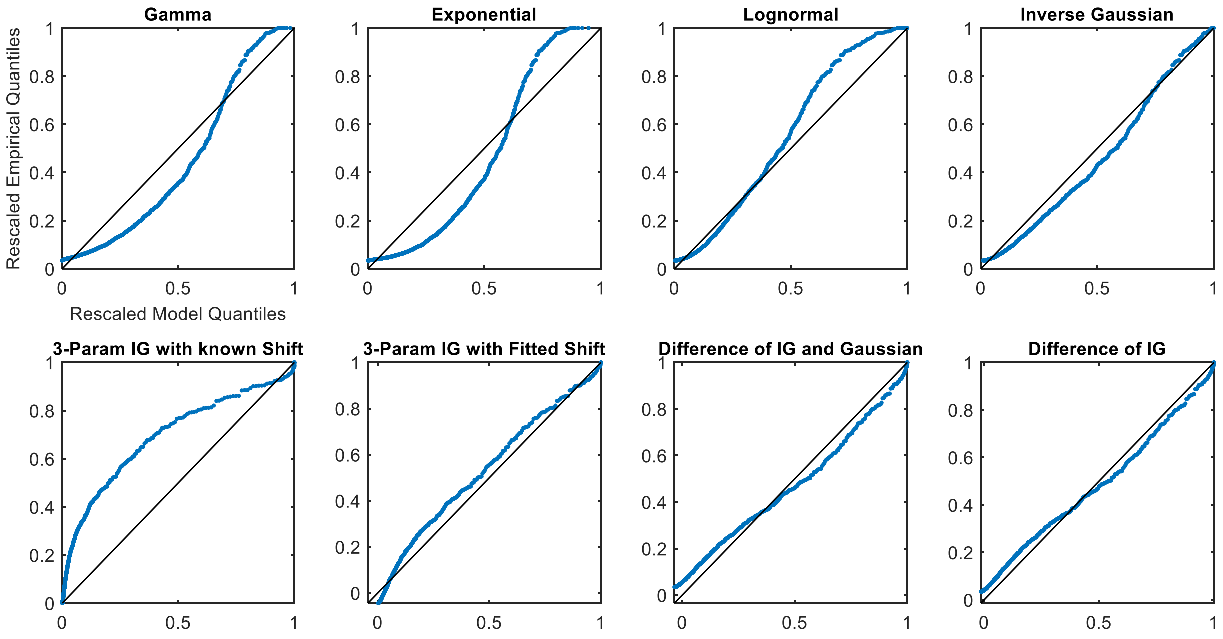

Fig AH. Rescaled QQ plots for Subject P7 from the propofol sedation cohort

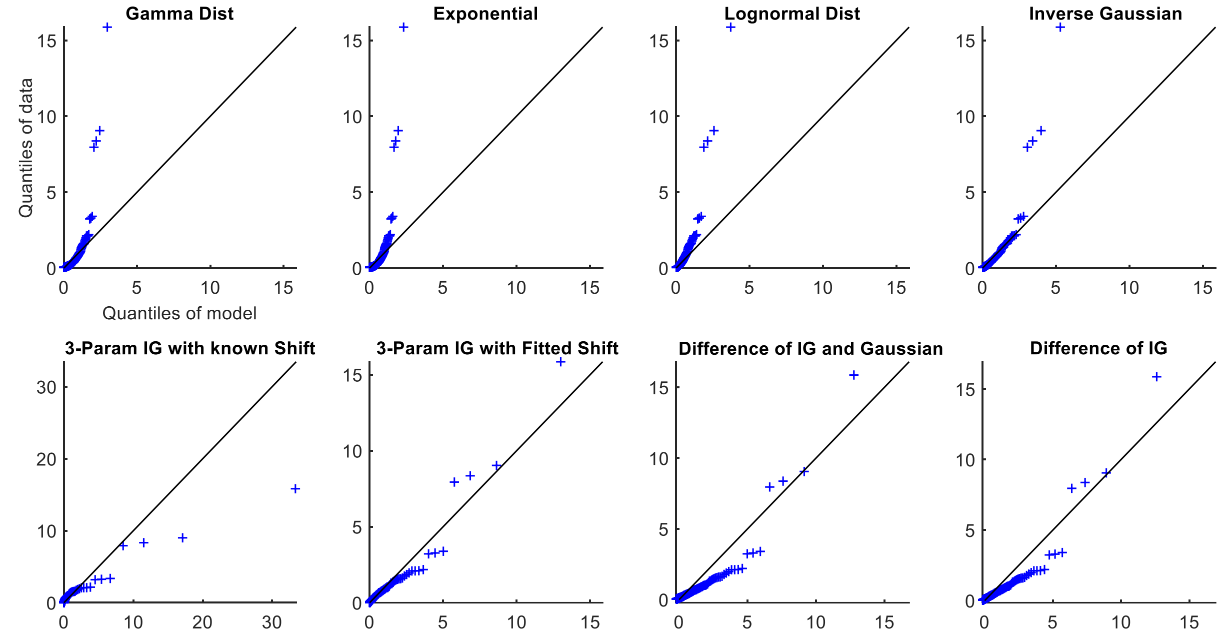

Fig AI. QQ plots for Subject P8 from the propofol sedation cohort

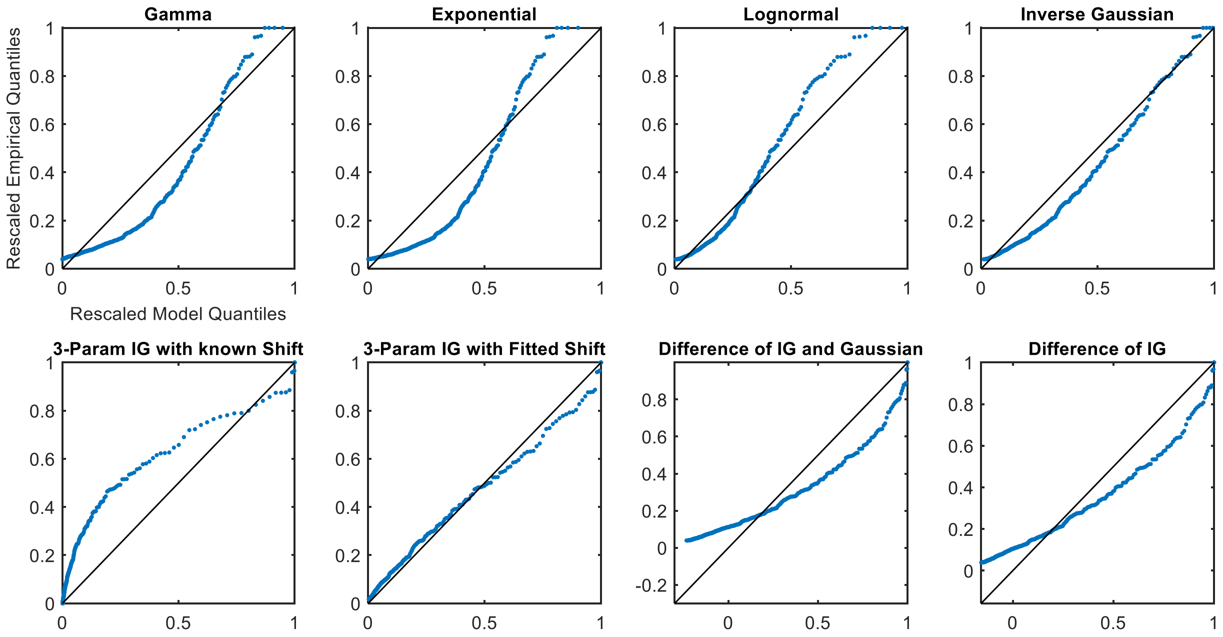

Fig AJ. Rescaled QQ plots for Subject P8 from the propofol sedation cohort

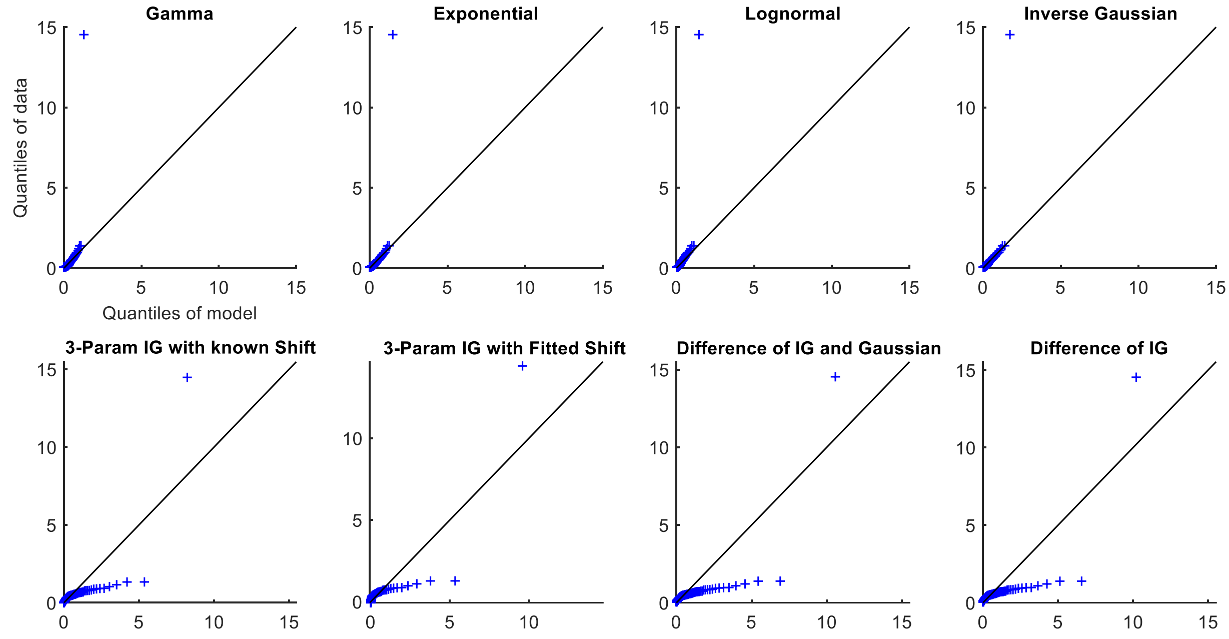

Fig AK. QQ plots for Subject P9 from the propofol sedation cohort

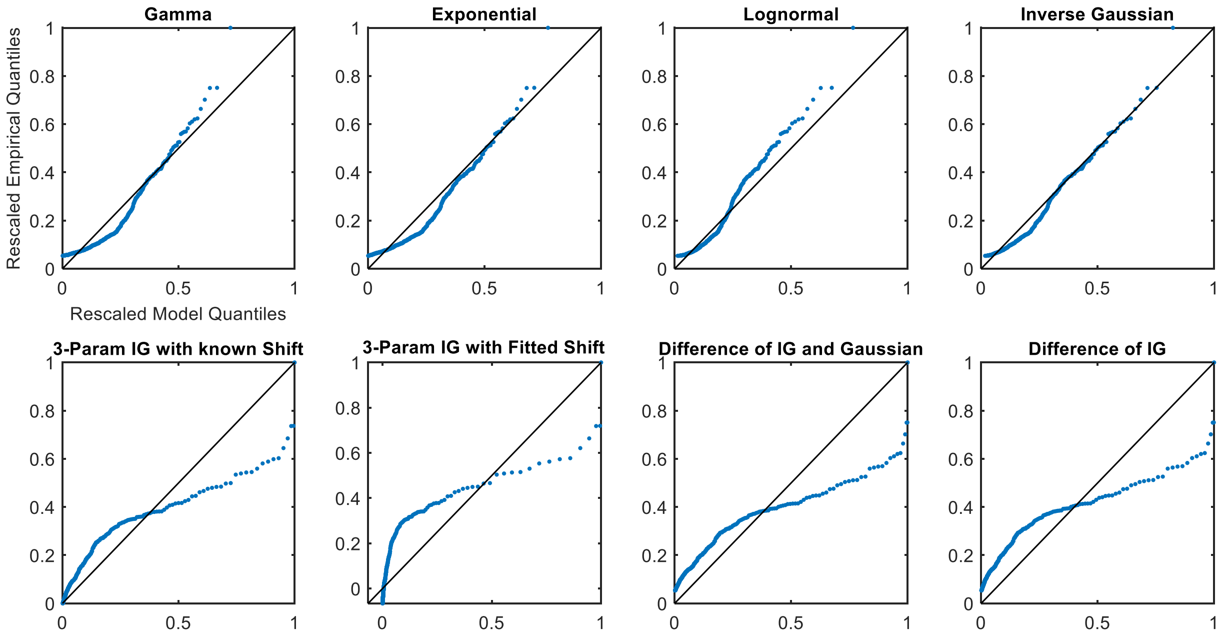

Fig AL. Rescaled QQ plots for Subject P9 from the propofol sedation cohort

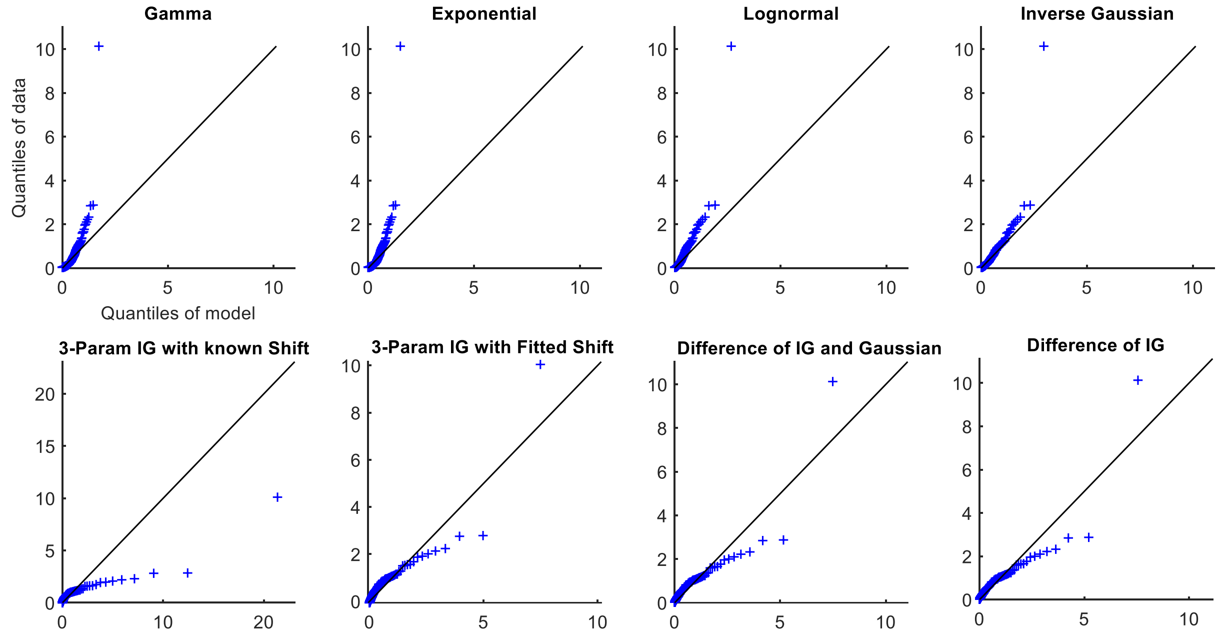

Fig AM. QQ plots for Subject P11 from the propofol sedation cohort

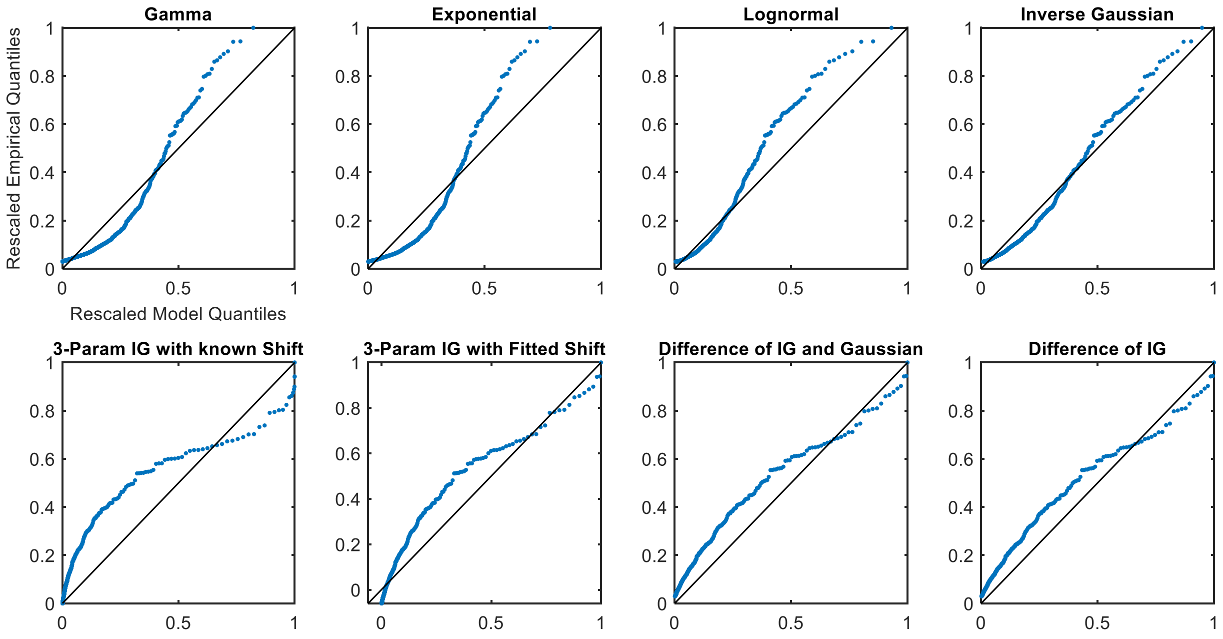

Fig AN. Rescaled QQ plots for Subject P11 from the propofol sedation cohort
