# Supplementary material for: Elementary integrate-and-fire process underlies pulse amplitudes in Electrodermal activity
Source: PLoS Comput Biol. 2021 Jul 7;17(7):e1009099. doi: 10.1371/journal.pcbi.1009099 (PMC8289084; doi:10.1371/journal.pcbi.1009099)
Supplement: S2 Appendix — (PDF) [file pcbi.1009099.s002.pdf]

S2 APPENDIX

Title: Elementary Integrate-and-Fire Process Underlies Pulse Amplitudes in Electrodermal Activity

Authors: Sandya Subramanian*, Patrick L. Purdon, Riccardo Barbieri, Emery N. Brown

*Corresponding Author

Corresponding Author E-mail: sandya@mit.edu

Detailed Anatomy and Physiology

An eccrine sweat gland has three parts: the dermal gland which secretes sweat, the duct which connects the gland to the surface of the skin, and the pore which opens the duct to the skin surface [1]. The dermal gland is innervated by a peripheral sympathetic nerve called the sudomotor nerve. Sympathetically-induced increases in spiking activity in the sudomotor nerves, called sudomotor bursts, cause sweat production in the gland. Sweat produced in response to these bursts accumulates in the duct, rising up to the skin surface by pushing open the pore. At the same time, sweat dissipates by constant reabsorption through the walls of the duct and by evaporation from the skin surface [1].

Sweat increases the skin's electrical conductance because the salt-containing sweat in the gland creates a low-resistance path through the skin, especially once it has crossed the high-resistance top layer of skin, the stratum corneum [1]. The greater the number of sweat glands bursting simultaneously, the greater the increase in conductance of the skin. The electrical conductance across the skin can be measured by placing two electrodes on the palmar surface of the hand, applying a constant voltage, and measuring the current [1]. The pulsatile changes in conductance measured in the skin are termed galvanic skin responses (GSRs).

References

1. Sonner Z et al. The microfluidics of the eccrine sweat gland, including biomarker partitioning, transport, and biosensing implications. Biomicrofluidics. 2015;9.
